# The Way Forward on Nutrition in Food Systems Transformation: A Response to the Recent Commentaries

**DOI:** 10.34172/ijhpm.2022.7797

**Published:** 2022-12-05

**Authors:** Gloria Cervantes, Carolina Pérez-Ferrer, Anne-Marie Thow, Eduardo Villarreal, Luis Durán-Arenas

**Affiliations:** ^1^Master´s and Doctorate Program in Medical and Health Sciences, Faculty of Medicine, National Autonomous University of Mexico, Mexico City, Mexico.; ^2^Center for Research in Nutrition and Health, National Institute of Public Health, Mexico City, Mexico.; ^3^National Council for Science and Technology (CONACYT), Mexico City, Mexico.; ^4^Menzies Centre for Health Policy, School of Public Health, The University of Sydney, Sydney, NSW, Australia.; ^5^Public Administration Division, Center for Research and Teaching in Economics, Mexico City, Mexico.; ^6^Public Health Department, Faculty of Medicine, National Autonomous University of Mexico, Mexico City, Mexico.

 We appreciate the interest in our recent paper and the two thoughtful commentaries that were published in this journal by Carriedo et al^1^ and Phulkerd.^2^ In this correspondence, we reflect upon both commentaries and discuss important elements brought by their authors for food system transformation, specifically: (*i*) power dynamics, (*ii*) policy coherence, and (*iii*) governance, with a systems thinking approach to progress on nutrition policy.

 We agree with Carriedo et al, that the role of *power dynamics* and corporate determinants of health (and food systems) must be addressed for food system transformation. We recognize that power is unequally distributed, globally and within countries, and that corporate interests have historically interfered with health policy efforts for better nutrition. There is no reason to believe that the substantive policies needed to improve food systems would be unchallenged by corporate interests. While we did not discuss corporate determinants of health (and food systems) in detail in our paper,^3^ we believe much can be done even within the constraints of the current economic system and current power dynamics.^4^ Academia and civil society groups have a crucial role to play in this attempt to rebalance power in favor of public health nutrition and the environment. In recent years, there are good examples of academia and civil society groups putting nutrition issues on the public agenda and successfully advocating for policy change. One example is how the food system transformation discussion started when academia and civil society actors engaged policy leaders from different sectors in the presentation of the book “*La Obesidad en México [Obesity in Mexico]*”^5^ As was said in one of the interviews^3^ with a Mexican stakeholder working in health:

 “*I met him (agriculture actor) for the first time in the presentation of the book. We had never worked together, but the conversation we had that day motivated us to work together and to align [health] objectives between our different government departments. That led us to establish the Intersectoral Group on Health and Food, Environment and Competitiveness (GISAMAC)” *[Government, Health actor].

 Carriedo et al also noted that despite power dynamics constraining policymaking, Mexico has been progressive with its food policy to target consumption of ultra-processed food. We agree with them, and believe that using that learning, we must anticipate some of the arguments that will be used by corporations to avoid the changes that are needed for food system transformation. For example, the argument of freedom of information and alignment to international standards. Corporations have argued that freedom of information initiatives which require greater nutritional reporting can discriminate against certain products without conveying complete information to consumers.^6^ They have also argued that (less rigorous) existing international standards should be used instead of raising local regulations.^6^ One challenge in food policy is to engage all stakeholders with a mechanism that balances power dynamics. We emphasize that food policy has to be the result of balance between the different actors and interests. Extreme positions can be of limited impact because of conflict and lack of commitment.

*Policy coherence* is another important element on food system transformation mentioned in the commentaries. Some barriers to policy coherence mentioned are political silos and the socio-economic and historical context. We acknowledge this difficulty in our paper where we reported that the GISAMAC had differences in perspectives from actors representing different sectors of interest (health, agriculture, trade) in the food system.^3^ It is considered a driver of policy coherence to have a clear integrated objective, or shared understanding across actors with differences in perspectives in the respective food policy sectors.^7,8^ In addition, as we highlighted in our analysis, nutrition is not embedded across all food and health relevant policies in Mexico. However, policy coherence can be fostered through institutional change and the creation of platforms that enable the building of shared objectives and understanding – and as such, we would suggest that the formation of the new GISAMAC provides an opportunity to foster policy coherence across agriculture, trade and health sectors.

 On the way forward in practice, how do we progress in nutrition policy? In theory, multistakeholder *governance* sounds good. It is the international organizations that talk about this, but what is done within countries? The United Nations, the World Health Organization (WHO) and other international organizations recommend multistakeholder health governance, including the private sectors, to be part of policymaking.^9,10^ However, at the local level in Mexico, GISAMAC has excluded corporate actors from any activity in the group’s policymaking in an informal way. On the way forward, can we find legally binding mechanisms that address power imbalances seen by the influence of corporate actors? We must favor the integration of an appropriate balance of interests among all the participant actors.^11^

 Phulkerd makes a very important point by highlighting the value of complex systems thinking for food systems transformation. As they note, solutions to one issue may create new problems for another, and this is precisely how we ended up where we are. Policies implemented in the name of economic development, such as trade agreements, have created new problems elsewhere, ie, dietary transformations leading to obesity.^12^ Likewise, we must avoid simplistic policies which in the name of health and nutrition which may be damaging to peoples livelihoods and countries’ economies. Both direct and indirect costs of different solutions should ideally be quantified, or at least considered, on the different parts of the system. Only by adopting a systems thinking approach will we be able to select the best solutions for people and the planet.^13^

## Acknowledgements

 This manuscript is drawn from a doctoral dissertation being prepared for the Master’s and Doctorate Program in Medical and Health Sciences of the National Autonomous University of Mexico. In addition, Dr. Karen Englander an English-speaking science editor provided assistance with English language editing.

## Ethical issues

 Not applicable.

## Competing interests

 Authors declare that they have no competing interests.

## Disclaimers

 We warrant that this manuscript is the original work of the authors listed and has not previously been published. It is not submitted to any other journal at this time. We warrant that all of the authors have agreed to this submission.

## Authors’ contributions

 All of the authors listed made contributions to the manuscript and qualify for authorship, and no authors have been omitted. LDA directed the response and reviewed the manuscript critically. GC drafted the manuscript and coordinated revisions. CPF provided insight into scope, contributed to manuscript draft and reviewed the manuscript critically. AMT and EV reviewed the manuscript critically.

## Funding

 This work was supported by the National Council for Science and Technology (CONACYT) in Mexico [grant number CVU 806118].
